# Revisiting cardiovascular risk reduction in type 2 diabetes and dyslipidemia

**DOI:** 10.1016/j.ijcrp.2022.200141

**Published:** 2022-06-23

**Authors:** Phillip Lim, David Bleich

**Affiliations:** Rutgers New Jersey Medical School, 185 South Orange Avenue, MSB I-588, Newark, NJ, 07103, USA

**Keywords:** CVD risk Reduction, Phenotypes, Type 2 diabetes, Triglycerides, Coronary calcium score

## Abstract

Statin therapy has been a mainstay of cardiovascular disease (CVD) risk reduction for the past 20 years in type 2 diabetes management. Its application has been largely due to well-designed, randomized-control studies consistently showing 25–35% CVD risk reduction. However, the remaining 65–75% reduction potential for CVD risk has yet to be effectively addressed. With a push towards personalized medicine, the likelihood of a one-size-fits-all approach to CVD risk reduction in type 2 diabetes may not be as beneficial as anticipated. It is reasonable to suggest that we have aggregated separate CVD phenotypic groups under one treatment umbrella and consequently, dismissed further unaddressed CVD risk reduction potential.

The hypothesis proposed in this review is that there are at least two phenotypic groups with distinct molecular mechanisms contributing to CVD risk requiring different treatment approaches that can be applied with present pharmacotherapy. The two phenotypes can be classified as the following: 1) high low-density lipoprotein (LDL) phenotype and 2) high triglyceride (TG) plus low high-density lipoprotein (HDL) phenotype. As both phenotypes are significantly represented in individuals with type 2 diabetes, a more precise understanding of molecular details can be merged with clinical CVD outcome studies to arrive at a new hypothesis for CVD treatment that can be substantiated with additional well-designed clinical trials. As we transition from 20th to 21st-century medicine, we should utilize new knowledge to adapt current CVD risk reduction measures for those with type 2 diabetes.

## 1 Introduction

Approximately 34 million Americans greater than 18 years of age (10.5%) have type 2 diabetes whereas more than 120 million Americans (48.0%) older than 20 years will ultimately develop cardiovascular disease [1,2]. Furthermore, cardiovascular disease (CVD) remains the leading cause of morbidity and mortality in patients with diabetes mellitus type 2 (T2DM) [[3], [4], [5]]. Over the past 30 years, the reduction of low-density lipoprotein (LDL) levels with statin drugs and the more recent proprotein convertase Subtilisin/Kexin type 9 serine protease (PCSK-9) inhibitors provided us with a promising direction for CVD risk reduction in T2DM. In major randomized placebo-controlled, double-blinded CVD studies, LDL lowering has conferred approximately 30% risk reduction for major adverse cardiovascular events (MACE) [6]. However, there is still approximately 70% remaining CVD risk reduction potential that is left unaddressed.

The present treatment strategy focused on statin therapy is not fundamentally flawed, but in 2021 it appears that we may have reached the limit of maximizing CVD risk reduction in those with T2DM and dyslipidemia. Furthermore, we have concluded that raising high-density lipoprotein (HDL) cholesterol with niacin or HDL-targeted drugs does not yield additional cardiovascular risk reduction [7,8]. Although we have data confirming the efficacy and inefficacy of multiple pharmaceutical agents regarding CVD risk reduction, is it possible that we have reached these conclusions while assuming T2DM comprises only one effective phenotype?

The purpose of this review is to define two well-recognized phenotypes of CVD in T2DM and to elucidate aspects of these phenotypic profiles regarding molecular pathophysiology and relevant clinical trial data. Finally, we aim to suggest personalized treatment strategies based on these two well-known phenotypes.

## 2 Methods

We performed a literature search on PubMed and Google Scholar of the following: statin therapy, fibrate therapy, cardiovascular risk reduction, hyperlipidemia, dyslipidemia, hypertriglyceridemia, type 2 diabetes, atherogenesis, low-density lipoprotein, high-density lipoprotein, lipoprotein lipase, triglycerides, coronary artery disease, coronary artery calcium scores, statin-induced myopathy, and nutraceuticals for cardiovascular disease reduction. The clinical aspects of dyslipidemia and its relevance regarding cardiovascular risk reduction through large randomized clinical trials were compared and analyzed. In addition to the study results, the strengths and limitations of these large, randomized lipid-lowering trials were utilized to propose the presence of multiple lipid phenotypes that could explain the unaddressed CVD risk reduction potential.

### 2.1 The high LDL phenotype and its atherogenic propensity

The first T2DM phenotype is represented by the high LDL profile. In the United States, more than 100 million, or approximately 53% of adults, have elevated serum LDL levels**.** [9] LDL was first discovered by John Gofman at the University of California Berkeley who found elevated LDL levels in the plasma of patients who suffered heart attacks [10]. Further studies of this correlation elucidated theories on the atherogenic effects of LDL by the binding of LDL to glycosaminoglycans in the arterial endothelium where the apoB molecule on LDL becomes modified via nearby oxidation reactions [11]. The modified apoB molecule triggers inflammation via macrophage recruitment and release of cytokines leading to arterial intima proliferation and the formation of atherosclerotic plaque [[12], [13], [14], [15]].

Patients with the high LDL phenotype typically have problems recycling LDL receptors in the liver. Westernized diets, contribute to excess cholesterol and consequent LDL elevation but appears to have profound adverse cardiovascular effects through non-LDL-related mechanisms. We indirectly see this evidence in studies like the Mediterranean Diet Study where the quality of dietary components (extra virgin olive oil or nuts) counted more towards decreased CVD risk than did reduction of LDL cholesterol levels [16]. In fact, LDL cholesterol levels did not appreciably decrease even though CVD risk was reduced by 31% in the Mediterranean diet with extra virgin olive oil group and 28% in the Mediterranean diet nut group. This CVD risk reduction was statistically significant and comparable to that seen in statins studies.

Here, approximately 70% of the study population had dyslipidemia as defined by LDL cholesterol >160 mg/dl or HDL level <40 in men and <50 in women. Moreover, approximately 50% of study subjects had type 2 diabetes. Indeed, the investigators intermingled more than one lipid phenotype, as we define in this manuscript, and ended up with excellent results. These results suggest that statin-independent mechanisms can have a profound effect on CVD endpoints. We will discuss later the possible reasons why these results were so impactful.

### 2.2 The high triglyceride and low HDL phenotype

The high triglyceride plus low HDL phenotype in insulin resistance was first explained by McGarry at University of Texas Southwestern Medical Center [17]. In their original publication, they described this phenotype as a consequence of insulin resistance in the liver. High levels of insulin impair lipoprotein lipase (LPL) activity by increasing the level of inhibitory apoprotein C-III (Apo C-III). Consequently, impaired LPL activity leads to increased triglycerides in the VLDL particle which then forms small dense LDL and HDL particles. These dense lipoproteins are highly atherogenic that can be readily oxidized.

Scrolling forward 30 years, we now understand through genome-wide association studies (GWAS) that the so-called “metabolic syndrome” phenotype of insulin resistance, high triglycerides, and low HDL, is associated with single nucleotide polymorphisms (SNPs) that prevent peripheral fat cell expansion in the hips and buttocks [18]. Fat ends up in visceral sites like the liver, mesentery and perhaps the coronary arteries thereby promoting inflammation. This discovery leads us back 50 years to phenotypic descriptions of individuals who possessed the so-called “apple” phenotype (fat that is stored in the mid-section and bust) in comparison to the so-called “pear” phenotype of individuals who stored fat in the hips and buttocks [19]. Those individuals with the “apple” phenotype were considered to have bad cardio-metabolic fitness, while those with the “pear” phenotype had good cardio-metabolic fitness.

### 2.3 High triglyceride levels can add CVD risk independently of LDL levels

Controversy has existed for the past 30 years concerning triglyceride reduction and cardiovascular risk. These are unfortunate consequences of statin-centric pharmaceutical studies as well as triglyceride studies with problematic study designs and underpowered data sets. The original Helsinki Heart Study (HHS) published in 1987 was a placebo-controlled, double-blinded study of 5081 Finnish men who were randomized to receive either placebo or gemfibrozil 600 mg twice daily over a 5-year period [20].

The cohort at randomization was predominantly non-diabetic (approximately 2.5% with diabetes) with a mean triglyceride level of approximately 175 mg/dL in both placebo and experimental groups. Mean triglyceride levels fell to 102.7 mg/dl (43% reduction) by year 1 in the gemfibrozil group and a sustained reduction of approximately 35% at the end of 5 years. The placebo group did not have any reduction in triglyceride levels throughout the study. Importantly, total cardiac events (fatal and non-fatal myocardial infarcts) decreased 34% in the gemfibrozil treated group (two-tailed p < 0.02) with no overall reduction in mortality. These results had blunted enthusiasm due to lack of reduced mortality, but it should be noted that there was an overall small number of mortalities in the study with the gemfibrozil group having 45 deaths and the placebo group having 42 deaths.

In contrast to the HHS, the Veterans Administration High-Density Lipoprotein Intervention Trial (VA-HIT) study showed a statistically significant association of baseline triglyceride levels with non-fatal MI and mortality associated with coronary heart disease (CHD) [21]. The VA-HIT Study, published in 2001, was a multi-centered, placebo-controlled, double-blinded study of 2531 men with known CAD and low HDL. Of those enrolled, 25% had diabetes at baseline. Patients were randomized to receive either placebo or gemfibrozil 600 mg twice daily over a 5-year period.

The primary aim of this study was to determine if raising HDL-cholesterol with gemfibrozil decreased coronary heart disease (CHD). Of note, patients were selected to have relatively low LDL cholesterol (∼111 mg/dl in both groups) and modestly elevated triglycerides (151 mg/dl at baseline). Mean HDL cholesterol at baseline was 31.5 mg/dl. While the mean triglyceride level fell to 101 mg/dl in the gemfibrozil group, only the increase in mean HDL cholesterol level to 33.4 mg/dl was associated with decreased CHD events. Moreover, baseline triglyceride levels had a significant association with CHD reduction especially at the highest tertile of triglyceride level (>180 mg/dl). Here, the relative CHD risk reduction with gemfibrozil was 28% in the VA-HIT study.

These findings concurred with a prior study called the Bezafibrate Infarction Prevention Study (BIPS) that showed 39.5% (p = 0.02) relative risk reduction of CHD for study subjects with baseline triglyceride levels ≥200 mg/dl over a 6.2-year study period [22]. This was a secondary prevention study of individuals with prior history of CAD. It should be noted that mortality rates and CHD event rates were the same in the overall study cohort that had a mean baseline triglyceride level of 145 ± 51 mg/dl for both study and placebo groups [22].

Since study results from statin trials were more consistent regarding CHD risk reduction and overall mortality, scientific interest and pharmaceutical drug development turned away from triglyceride-lowering studies until the FIELD study, published in 2005 [23]. The FIELD (fenofibrate intervention and event lowering in diabetes) study randomized ∼4900 study participants with type 2 diabetes to either placebo or fenofibrate and followed them for 5 years. Of important note, it was determined over the course of the study that 17% of placebo-treated study subjects compared to 8% of fenofibrate-treated subjects were taking newly prescribed statin drugs (p < 0.0001) [23]. Concern was raised about the study design and ability to interpret study data due to the high “contamination” rate of statin usage in the placebo group. This might have inadvertently lowered the CVD event rate in this group and thereby reduced the apparent efficacy of fenofibrate. Notwithstanding, fenofibrate was associated with an 11% relative reduction in CVD events compared to placebo. This 11% reduction in CVD events was not statistically significant [23].

More recently, the ACCORD lipid study published in 2010 compared simvastatin alone (placebo group) versus simvastatin plus fenofibrate (experimental group) in a cohort of 5500 individuals with type 2 diabetes [24]. After 8 years, the experimental group showed no difference in major adverse cardiovascular events compared to the placebo. One might think that this study ended the debate concerning fibrate treatment for individuals with type 2 diabetes and the metabolic syndrome lipid profile (High triglyceride plus low HDL). Unfortunately, the mean triglyceride in the study cohorts was only 162 mg/dl; therefore, no conclusion could be drawn about a more relevant sub-group of individuals with higher baseline triglycerides. Indeed, when subgroup analysis was performed on the Accord Lipid Study cohort, a highly significant CVD risk reduction of 31% was revealed in the fibrate-treated group [25]. Unfortunately, this was a sub-group analysis which is not powered to make cause and effect statements from study data.

### 2.4 Lowering triglyceride levels account for additional CVD risk that is unmet by statin treatment

**Fig. 1:**
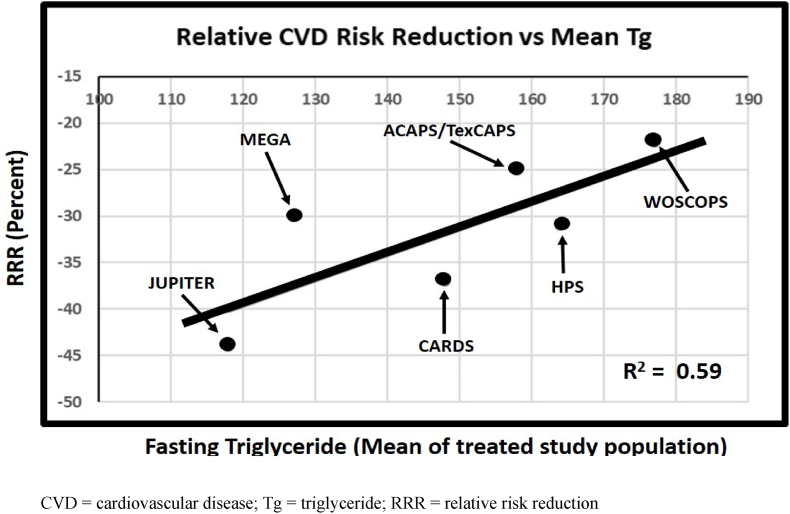
CVD = cardiovascular disease; Tg = triglyceride; RRR = relative risk reduction.

Studies have shown that statins reduce cardiovascular risk in type 2 diabetes by 25–35% on average. Further reduction of LDL to approximately 50% of baseline levels can be achieved with PCSK-9 inhibitors. However, while ultra-low LDL levels decreased myocardial infarction by approximately 28%, it did not affect all-cause mortality or cardiovascular mortality except in patients with higher baseline LDL levels [26]. Where then is the unaccounted cardiovascular risk if not from LDL lowering? In order to explore an answer, linear regression analysis was performed on the major published primary cardiovascular risk reduction studies using statins (Fig. 1).

As shown below, when plotting CVD risk reduction in major primary CVD prevention trials against baseline triglyceride level in the select treatment cohorts we recognize a highly relevant coefficient of determination (r^2^ = 0.59; p < 0.04). Indeed, those study groups with higher mean triglyceride levels at baseline had lower CVD risk reduction with statin treatment. Moreover, the relative LDL reduction was similar in these plotted studies. This analysis allows us to propose the hypothesis that baseline triglyceride level contributes to cardiovascular risk independent of LDL lowering with statins. It also supports the fundamental notion that there may be at least two distinct phenotypes of individuals with type 2 diabetes who require different treatment algorithms to attenuate CVD risk.

The notion that additional CVD risk reduction can be achieved beyond statin treatment was finally demonstrated in the Reduce-IT Study. Here, eicosapentaenoic acid (EPA) was given with simvastatin in the experimental group, while the control group received simvastatin only. This is the first large double-blinded, randomized, placebo-control study to show additional risk reduction beyond that attainable with a statin. It is worthwhile to note that using a statin to reduce ox-LDL in combination with EPA to saturate the lipid scavenging receptor CD36 addressed two potential mechanisms responsible for vascular endothelial dysfunction. Moreover, basic research studies demonstrate that EPA included in the diet of older rats was able to reverse vascular endothelial dysfunction by improving endothelial-dependent eNOS relaxation and normalizing expression of ACE and angiotensin-1 receptor expression [27].

Total enrollment in the Reduce-IT study was approximately 4000 high-risk subjects in each group with a study duration of 5 years. Patients received 2 g of EPA twice daily or a mineral oil placebo that mimicked the visible characteristics of EPA. Baseline triglyceride levels were 216.5 mg/dl in both groups with approximately 60% having diabetes and approximately 93% previously treated with moderate or high dose statin. Average triglyceride-lowering after 1 year was −39.0 mg/dl in the experimental group and +4.5 mg/dl in the placebo group. Overall CVD risk reduction was 26% in the group receiving EPA.

Importantly, a statistically significant difference in risk was seen in study subjects with baseline fasting triglyceride level ≥150 mg/dl, but not those with levels <150 mg/dl. This observation is once again consistent with prior studies showing significant CVD risk reduction when triglyceride levels are decreased from “significantly” elevated levels.

### 2.5 Personalized treatment for CVD risk reduction in type 2 diabetes

Most patients with type 2 diabetes demonstrate the phenotypic attributes of metabolic syndrome (MS). In one study that followed approximately 3300 subjects for 8 years, approximately 60% of men and 50% of women had criteria of MS. Indeed, when MS was present, the mean baseline fasting triglyceride levels for men and women were 208 ± 114 mg/dL and 187 ± 117 mg/dL, respectively. Those individuals without MS had fasting triglyceride levels of 106 ± 71 and 87 ± 43. HDL cholesterol was similarly affected by MS in both sexes [28]. These two different phenotypes (high LDL versus high triglyceride/low HDL) present us with the opportunity to personalize treatment strategies for dyslipidemia in individuals with type 2 diabetes. So, what does CVD risk reduction look like for individuals with type 2 diabetes and only high LDL; high triglyceride plus low HDL; or mixed dyslipidemia (both high LDL and triglycerides plus low HDL)?

If we use the Heart Risk Calculator (http://www.cvriskcalculator.com/) that follows the American College of Cardiology/American Heart Association guidelines updated in 2017 with several assumptions, we can make reasonable estimates of CVD risk and relative risk reduction in type 2 diabetes using different scenarios and best available study data. Importantly, there are limitations to the imputed data including age restriction between 40 and 79 years, total cholesterol ≤320 mg/dL, and the lack of consideration of fasting triglyceride levels. Nevertheless, the risk calculator can be used to estimate primary CVD risk reduction with a statin or PCSK-9 inhibitor treatment, thereby allowing an approximate comparison of different treatment modalities. Additionally, to determine CVD risk reduction with fibrate treatment in those individuals with the high triglyceride/low HDL or mixed phenotypes, it is necessary to reference the HHS which studied middle-age Scandinavian males. These results might not apply to other ethnic groups or women.

**Fig. 2:**
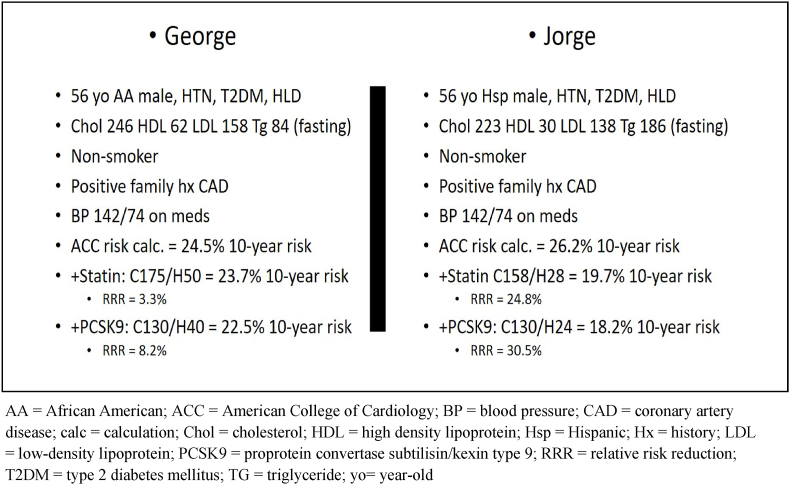
AA = African American; ACC = American College of Cardiology; BP = blood pressure; CAD = coronary artery disease; calc = calculation; Chol = cholesterol; HDL = high density lipoprotein; Hsp = Hispanic; Hx = history; LDL = low-density lipoprotein; PCSK9 = proprotein convertase subtilisin/kexin type 9; RRR = relative risk reduction; T2DM = type 2 diabetes mellitus; TG = triglyceride; yo = year-old.

For comparison, let's evaluate two hypothetical case studies George and Jorge. These are two 56-year-old males with high-risk CVD profiles (Fig. 2). George is an African American male who represents the high LDL phenotype, while Jorge is a Hispanic male with the mixed phenotype. Both have estimated 10-year ACC risks of ∼25% due to the male gender, age, diabetes, and hypertension. Of particular interest, George only gets a 3.3% relative risk reduction with high-dose statin treatment that decreases his total cholesterol from 246 to 175 mg/dL (28.9% relative decrease in cholesterol level). The addition of a PCSK-9 inhibitor provides an additional CVD risk reduction of ∼5% by reducing his total cholesterol to 130 mg/dl (LDL cholesterol is not used in the calculator, but we can assume that LDL will be proportionately decreased as total cholesterol). George's LDL cholesterol would therefore be reduced by ∼47% with statin + PCSK-9 inhibitor or 84 mg/dl, not quite at goal <70 mg/dl.

In comparison, Jorge has a much better reduction in CVD relative risk with a statin (∼29.1% decrease in total cholesterol), even though he has a similar overall profile as George. Here, Jorge obtains a 24.8% relative CVD risk reduction with a high-dose statin even though he has a similar percent reduction in total cholesterol (Jorge −29.1% versus George −28.9%). The addition of a PCSK-9 inhibitor gives Jorge a total CVD risk reduction of 30.9% with ∼42% reduction in total cholesterol (Jorges’ LDL would be ∼80 mg/dl). Why does Jorge benefit so much more from statin treatment than George? The answer is not exactly clear, but certainly, race is one factor.

A second factor is that the low HDL phenotype receives negative points in CVD risk reduction. Thus, a part of the high triglyceride/low HDL phenotype is accounted for in a standard CVD risk score. Finally, statin treatment indirectly lowers VLDL triglyceride by interfering with cholesterol biosynthesis. It is also important to recognize that there are no approved CVD risk calculators to date that include triglyceride levels.

What would happen to Jorge's CVD risk score if we treated him with gemfibrozil first? Using HHS data to assume CVD risk reduction, we would expect a 34% relative CVD risk reduction in coronary heart disease (not mortality) over 5 years. Fibrate treatment would translate into the following lipid profile for Jorge: cholesterol 225, HDL 31, and triglyceride 126. It appears that George would do better with LDL lowering while Jorge would do as well or better with triglyceride-lowering if we assume maximal LDL reduction with statin + PCSK-9 inhibitor.

The comparison of George and Jorge reveals to us that a “one-size-fits all” approach to CVD risk reduction may be imprecise. New insights about the molecular pathophysiology of CVD uncover unattributed CVD risk in type 2 diabetes and this brings us back to the Reduce-It Study.

### 2.6 The Reduce-It Study

Here, it is important to compare EPA with our previous treatment for CVD risk with fish oil capsules. As demonstrated by the VITAL Research Group, 1 g per day of marine n-3 fatty acid did not lower CVD risk; only 13% of the study group had diabetes [29]. Fish oil composition is highly variable depending on species and habitat and contains docosahexaenoic acid (DHA), linoleic acid (LA), and eicosapentaenoic acid (EPA), as well as other fatty acids.

One study quantified polyunsaturated fatty acid composition from tuna oil as 70.2% DHA, 22.4% EPA, and 2.1% LA [30]. Another study used fish oil from multiple species that was a byproduct of industrial-scale processing. Here, the composition of the 3 key PUFAs were LA 17.7%, DHA 5.9% and EPA 4.0% [31]. The importance of this information is that EPA tends to be a minor oil component of fish oil, yet it has robust anti-inflammatory properties when compared to LA and DHA. This insight was exploited in the Reduce-IT Study that led to excellent reduction in CVD with EPA.

### 2.7 Heterogeneity in those eligible for statin therapy

The existence of multiple lipid phenotypes and their respective mechanisms for lipid lowering therapy is supported by the MESA (Multi-ethnic Study of Atherosclerosis) study. Of the 4758 participants, 2377 (50%) were recommended moderate-to-high intensity statin therapy based on a 10-year estimated ASCVD risk of greater than or equal to 7.5% [32]. Of those who were recommended statins, 41% had a coronary artery calcium (CAC) score of 0, suggesting a potential underlying heterogeneity in lipid phenotypes linked to CVD risk [32]. The implication of the MESA study is that the classic indicators of CVD risk (hypertension, cholesterol, HDL, ethnicity, sex, diabetes, and cigarette smoking) do not correlate well with anatomic evaluation of the coronary arteries (CAC score). This discordance provides us with an opportunity to combine these biomarkers into a more robust risk calculator.

## 3 Discussion

### 3.1 Multiple lipid phenotypes

We propose two lipid phenotypes in those with diabetes and the potential for further CVD risk reduction with triglyceride lowering therapy. By no means is this an exhaustive representation of possible phenotypes. Nonetheless, it highlights the importance of utilizing a more individualized approach in order to maximize CVD risk reduction, especially in patients with diabetes. There is ample evidence to support the concept of triglyceride reduction in diabetic patients with high triglyceride and low HDL for CVD risk reduction. One question would be how high to set the fasting triglyceride threshold to achieve CVD risk reduction. Since most studies have shown CVD risk reduction above 150 mg/dL, we propose setting a fasting triglyceride level above 160 mg/dL as the lower threshold to treat with fibrate drugs such as gemfibrozil, bezafibrate, or fenofibrate at a maximally tolerated dose with or without a statin drug.

Alternatively, those individuals within the high LDL phenotype should receive statin treatment for primary and secondary prevention to lower LDL cholesterol to <70 mg/dL. If patients do not tolerate statins or do not get the necessary LDL reduction, then a PCSK-9 inhibitor can be introduced if possible. What about individuals who are unable to tolerate statin drugs?

### 3.2 Statin intolerance

Statin intolerance is yet another obstacle for lipid lowering therapy that affects lipid phenotypes differently. It is an area of research that has gained ground over the past several years and has shown a potential underestimation of this class of adverse drug effects. A recent meta-analysis of stain-intolerance reported ∼20% of individuals developing an “inability to tolerate at least two statins: one statin at the lowest starting daily dose and another statin at any daily dose, due to either objectionable symptoms (real or perceived) or abnormal laboratory determinations, which are temporally related to statin treatment and reversible upon statin discontinuation.” [33,34] Furthermore, the GAUSS3 study, a blinded randomized clinical trial, showed that 42.6% of patients reported intolerable muscle symptoms while on atorvastatin but not placebo [35].

Therefore, we have an important decision to make in mitigating CVD risk for statin-intolerant individuals with type 2 diabetes. Do we continue them on statin-therapy without regard to the symptoms? Do we discontinue statin treatment with the recognition of increasing CVD risk, or can we travel down an alternative therapeutic road? One solution is addition of PCSK-9 inhibitor, but this road is fraught with problems and often requires prior authorization or non-approval by insurance plans even with clear evidence of classic stain-induced myopathy.

### 3.3 The utility of coronary CT scan and beyond in profiling type 2 diabetes CVD risk

In populations that cannot tolerate statins, coronary calcium scores (CCS) may be a useful tool for the evaluating the indication of lipid lowering therapies. CCS have been around for about twenty years and diabetic subjects, in particular, were found to exhibit a 4–5 fold increased risk of CVD over a 10-year period when the scores are 0 versus ≥400 [36]. When viewed as a lifetable over 12.5 years, subjects with diabetes of any duration and CCS = 0 had ∼95% coronary heart disease survival, while those with scores ≥400 drop to 80% (diabetes <10 years) and 68% (diabetes ≥10 years) survival [36].

When compared to the gold standard coronary angiogram in patient >50 years of age, CCS had a sensitivity of 98–100% [37]. For statin-intolerant individuals with type 2 diabetes, a CCS = 0 gives the care provider a high degree of certainty that the is little likelihood of a coronary event and allows the provider confidence that stain treatment can be withheld.

### 3.4 Limitations

This manuscript is a review on current literature supporting multiple clinical subcategories of dyslipidemia in type 2 diabetes and the respective implications regarding cardiovascular risk reduction. While we have analyzed strengths and weaknesses of landmark studies regarding this topic, this manuscript does not represent a randomized clinical trial, retrospective analysis, cohort study, or a systematically approached meta-analysis. Although this review covers diet studies and their contribution to CVD risk reduction, we did not go into the specific nutraceuticals that have shown to significantly influence CV risk reduction [38]. Studies delving into nutraceuticals in the different dyslipidemia phenotypes of type 2 diabetic patients and their synergistic effects of reducing multiple CV risk factors may play also be an explanation for the unaccounted CVD risk reduction from pure statin therapy.

## 4 Conclusion

**Fig. 3:**
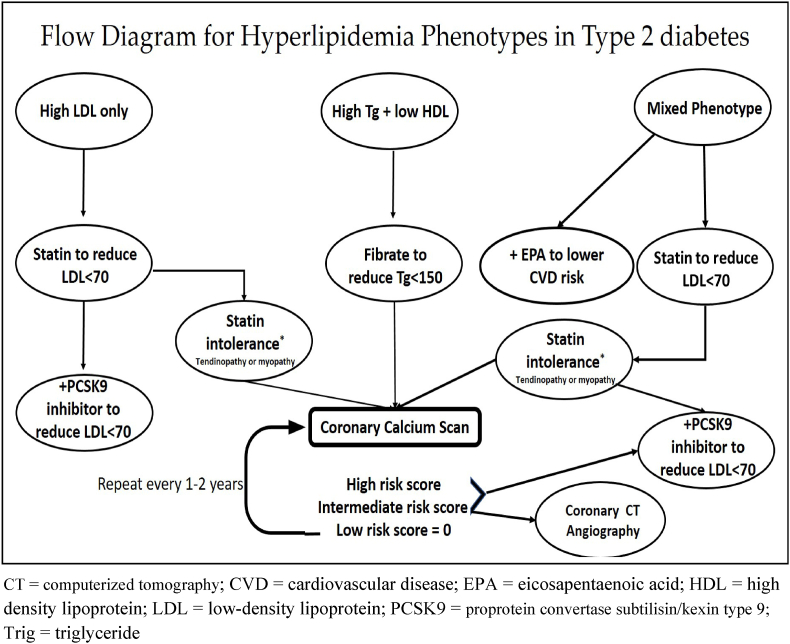
CT = computerized tomography; CVD = cardiovascular disease; EPA = eicosapentaenoic acid; HDL = high density lipoprotein; LDL = low-density lipoprotein; PCSK9 = proprotein convertase subtilisin/kexin type 9; Trig = triglyceride.

We propose at least two lipid phenotypes that highlight different mechanisms of CVD risk reduction outside of lowering LDL with statin therapy. As both phenotypes are significantly represented in individuals with type 2 diabetes, a more precise understanding of molecular details and clinical data is necessary for the development of individualized treatment. Until additional mechanisms and characteristics of lipid phenotypes are discovered, we propose a schematic flow diagram in Fig. 3 representing a new paradigm for individualized CVD risk reduction in patients with type 2 diabetes. While not all parts of this schema have been validated with randomized, double-blinded, placebo-controlled clinical trials, it provides a roadmap to personalized lipid management without restricting to only LDL lowering strategies. Future studies will need to validate new treatment paradigms as we move from personalized to precision medicine.

## Credit roles

Dr. Bleich formulated the idea, wrote the initial draft of the article, edited the article and final draft, and provided critical review. Dr. Lim revised the article, edited the article, and provided additional text and references to the original draft.

## Declaration of competing interest

Dr. Bleich has served as Principal Investigator for Bayer Pharmaceutical. Dr. Lim has nothing to declare.

## References

[1] (2020). National Diabetes Statistics Report.

[2] Virani S.S., Alonso A., Benjamin E.J., Bittencourt M.S., Callaway C.W., Carson A.P. (2020). Heart disease and stroke statistics-2020 update: a report from the American heart association. Circulation.

[3] American Diabetes A. (2020). 10. Cardiovascular disease and risk management: standards of medical care in diabetes-2020. Diabetes Care.

[4] Davies M.J., D'Alessio D.A., Fradkin J., Kernan W.N., Mathieu C., Mingrone G. (2018). Management of hyperglycemia in type 2 diabetes, 2018. A consensus report by the American diabetes association (ADA) and the European association for the study of diabetes (EASD). Diabetes Care.

[5] American Diabetes A. (2018). Economic costs of diabetes in the U.S. in 2017. Diabetes Care.

[6] Cheung B.M., Lauder I.J., Lau C.P., Kumana C.R. (2004). Meta-analysis of large randomized controlled trials to evaluate the impact of statins on cardiovascular outcomes. Br. J. Clin. Pharmacol..

[7] Investigators A.-H. (2011). Niacin in patients with low HDL cholesterol levels receiving intensive statin therapy. N. Engl. J. Med..

[8] Barter P., Caulfield M., MEriksson M., Grundy S.M., Kastelein J., Jomajda M. (2007). Effects of torcetrapib in patients at high risk for coronary events. N. Engl. J. Med..

[9] (2011 Feb 1). Vital Signs: Prevalence, Treatment, and Control of High Levels of Low-Density Lipoprotein cholesterol—United States, 1999-2002 and 2005-2008.

[10] Gofman J.W.D.O., Glazier F., Freeman N.K., Lindgren F.T., Nichols A.V., Strisower B., Tamplin A.R. (1954). The serum lipoprotein transport system in health, metabolic disorders, atherosclerosis and coronary heart disease. Plasma.

[11] Bonetti P.O., Lerman L.O., Lerman A. (2003). Endothelial dysfunction: a marker of atherosclerotic risk. Arterioscler. Thromb. Vasc. Biol..

[12] Steinberg D., Witztum J.L. (2010). Oxidized low-density lipoprotein and atherosclerosis. Arterioscler. Thromb. Vasc. Biol..

[13] Brown M.S., Goldstein J.L. (1983). Lipoprotein metabolism in the macrophage. Annu. Rev. Biochem..

[14] Hansson G.K., Jonasson L. (2009). The discovery of cellular immunity in the atherosclerotic plaque. Arterioscler. Thromb. Vasc. Biol..

[15] Libby P., Ridker P.M., Hansson G.K. (2011). Progress and challenges in translating the biology of atherosclerosis. Nature.

[16] Estruch R.R.E., Salas-Salvadó J., Covas M.I., Corella D., Arós F., Gómez-Gracia E., Ruiz-Gutiérrez V., Fiol M., Lapetra J., Lamuela-Raventos R.M., Serra-Majem L., Pintó X., Basora J., Muñoz M.A., Sorlí J.V., Martínez J.A., Fitó M., Gea A., Hernán M.A., Martínez-González M.A. (2018). PREDIMED study investigators. Primary prevention of cardiovascular disease with a mediterranean diet supplemented with extra-virgin olive oil or nuts. N. Engl. J. Med..

[17] McGarry J.D. (1992). What if minkowski had been ageusic? An alternative angle on diabetes. Science.

[18] Lotta L.A., Wittemans L.B.L., Zuber V., Stewart I.D., Sharp S.J., Luan J. (2018). Association of genetic variants related to gluteofemoral vs abdominal fat distribution with type 2 diabetes, coronary disease, and cardiovascular risk factors. JAMA.

[19] Bauer R.C., Khetarpal S.A., Hand N.J., Rader D.J. (2016). Therapeutic targets of triglyceride metabolism as informed by human genetics. Trends Mol. Med..

[20] Frick M.H.E.O., Haapa K., Heinonen O.P., Heinsalmi P., Helo P., Huttunen J.K., Kaitaniemi P., Koskinen P., Manninen V. (1987). Helsinki Heart Study: primary-prevention trial with gemfibrozil in middle-aged men with dyslipidemia. Safety of treatment, changes in risk factors, and incidence of coronary heart disease. N. Engl. J. Med..

[21] Robins S.J.C.D., Wittes J.T., Papademetriou V., Deedwania P.C., Schaefer E.J., McNamara J.R., Kashyap M.L., Hershman J.M., Wexler L.F., Rubins H.B., Study Group V.A.-H.I.T. (2001). Veterans Affairs High-Density Lipoprotein Intervention Trial. Relation of gemfibrozil treatment and lipid levels with major coronary events: VA-HIT: a randomized controlled trial. JAMA, J. Am. Med. Assoc..

[22] Infarction Prevention Study Bezafibrate (2000). Secondary prevention by raising HDL cholesterol and reducing triglycerides in patients with coronary artery disease. Circulation.

[23] Scott R., Best J., Forder P., Taskinen M.R., Simes J., Barter P. (2005). Fenofibrate Intervention and Event Lowering in Diabetes (FIELD) study: baseline characteristics and short-term effects of fenofibrate [ISRCTN64783481]. Cardiovasc. Diabetol..

[24] Group T.A.S. (2010). Effects of combination lipid therapy in type 2 diabetes mellitus. N. Engl. J. Med..

[25] Tenenbaum A., Fisman E.Z. (2012). Fibrates are an essential part of modern anti-dyslipidemic arsenal: spotlight on atherogenic dyslipidemia and residual risk reduction. Cardiovasc. Diabetol..

[26] Karatasakis A., Danek B.A., Karacsonyi J., Rangan B.V., Roesle M.K., Knickelbine T. (2017). Effect of PCSK9 inhibitors on clinical outcomes in patients with hypercholesterolemia: a meta-analysis of 35 randomized controlled trials. J. Am. Heart Assoc..

[27] Farooq M.A., Gaertner S., Amoura L., Niazi Z.R., Park S.H., Qureshi A.W. (2020). Intake of omega-3 formulation EPA:DHA 6:1 by old rats for 2 weeks improved endothelium-dependent relaxations and normalized the expression level of ACE/AT1R/NADPH oxidase and the formation of ROS in the mesenteric artery. Biochem. Pharmacol..

[28] Wilson P.W., D'Agostino R.B., Parise H., Sullivan L., Meigs J.B. (2005). Metabolic syndrome as a precursor of cardiovascular disease and type 2 diabetes mellitus. Circulation.

[29] Manson J.E., Cook N.R., Lee I.M., Christen W., Bassuk S.S., Mora S. (2019). Marine n-3 fatty acids and prevention of cardiovascular disease and cancer. N. Engl. J. Med..

[30] Suseno S., Hayati S., Izaki A. (2014). Fatty acid composition of some potential fish oil from production centers in Indonesia. Orient. J. Chem..

[31] Dobrzanksi Z., Bykowski P., Iwaniuk Z., Usydus Z., Gorecka H., Trziszka T. (2002). Evaluation of the chemical composition of fish oil: a by-product from fish processing plants. Bull. Sea Fisher. Inst..

[32] Nasir K., Bittencourt M.S., Blaha M.J., Blankstein R., Agatson A.S., Rivera J.J. (2015). Implications of coronary artery calcium testing among statin candidates according to American College of Cardiology/American heart association cholesterol management guidelines: MESA (Multi-Ethnic study of atherosclerosis). J. Am. Coll. Cardiol..

[33] Toth P.P.P., Angelo Maria, Giglio Rosaria Vincenza, Nikolic Dragana, Castellino Giuseppa, Rizzo Manfredi, Banach Maciej (2018). Management of statin intolerance in 2018: still more questions than answers. Am. J. Cardiovasc. Drugs.

[34] Jacobson T.A., Ito M.K., Maki K.C., Orringer C.E., Bays H.E., Jones P.H. (2014). National Lipid Association recommendations for patient-centered management of dyslipidemia: part 1 - executive summary. J. Clin. Lipidol..

[35] Nissen S.E., Stroes E., Dent-Acosta R.E., Rosenson R.S., Lehman S.J., Sattar N. (2016). Efficacy and tolerability of evolocumab vs ezetimibe in patients with muscle-related statin intolerance: the GAUSS-3 randomized clinical trial. JAMA.

[36] Malik S.Z.Y., Budoff M., Nasir K., Blumenthal R.S., Bertoni A.G., Wong N.D. (2017). Coronary artery calcium score for long-term risk classification in individuals with type 2 diabetes and metabolic syndrome from the multi-ethnic study of atherosclerosis. JAMA Cardiol..

[37] Lamont D.H., Budoff M.J., Shavelle D.M., Shavelle R., Brundage B.H., Hagar J.M. (2002). Coronary calcium scanning adds incremental value to patients with positive stress tests. Am. Heart J..

[38] Borghi C., Fogacci F., Agnoletti D., Cicero A.F.G. (2022). Hypertension and dyslipidemia combined therapeutic approaches. High Blood Pres. Cardiovasc. Prev..

